# Feasibility and Impact of Doctor-Nurse Task Delegation in Preventive Child Health Care in the Netherlands, a Controlled Before-After Study

**DOI:** 10.1371/journal.pone.0139187

**Published:** 2015-10-14

**Authors:** S. Janine Benjamins, Maurice L. W. Damen, Henk F. van Stel

**Affiliations:** 1 Icare JGZ, Blankenstein 400, P.O. box 900, 7940 KE, Meppel, the Netherlands; 2 Department of Health Technology Assessment, Julius Center for Health Sciences and Primary Care, University Medical Center Utrecht, P.O. box 85500, 3508 GA, Utrecht, the Netherlands; Centre Hospitalier Universitaire Vaudois, FRANCE

## Abstract

**Background:**

In the Netherlands a need is felt for more flexible Child Health Care services, both efficient and tailored to needs. We set up a study on impact and feasibility of task delegation to child health care nurses performing all regular checkups on children aged 2 months to 4 years. Abnormal findings were discussed with the attending child health care doctor. This article describes impact and feasibility of this task delegation from four viewpoints: competences of nurses; percentage of children assigned to the nurse; change in abnormal findings and referrals; experiences of professionals and parents.

**Methods:**

Two experiment teams and two control teams were compared before and after starting task delegation. Nurses in the experiment teams were trained to carry out regular checkups on healthy children. Assignment to the experiment schedule was a joint decision by doctor and nurse. Nursing competences were measured by means of questionnaires. Percentage of children assigned to the nurse and screening results of eyes, heart, hips, growth and development were extracted from the electronic health record. Difference in change was compared between experiment and control teams. Mann-Whitney tests and logistic generalized estimating equations were used to test for significance. Experiences of professionals and parents were evaluated through focus group interviews, which were subjected to a qualitative analysis.

**Results:**

Nurses in the experiment regions showed improvement in medical screening skills. No difference in change was perceived in general nursing competences. In the experiment group, 69% of all children were assigned to the nurse. There were no significant differences in change in the percentages of abnormal findings or referrals in the experiment teams compared to the control teams, except for hips. Interviews showed that both doctors and nurses thought positively of the new working method, yet made some recommendations for improvements. Parents felt well-informed and experienced an equal level of proficiency but less continuity in person.

**Conclusion:**

This experiment shows that task delegation from doctor to nurse in preventive child health care is feasible. It is important to pay attention to the acceptation process of professionals during implementation. More investigation is needed in order to assess effectiveness and efficacy of task delegation.

## Introduction

Traditionally, preventive child health care in the Netherlands is provided by medical doctors and nurses specialized in this area. During the first four years of their lives children are checked upon 15 times by a doctor or a nurse alternately. The main focus is on prevention and early detection of health problems through vaccinations, screening programmes and health advice. Society is changing however and so are the demands for care. First, psychosocial problems are becoming more and more of an issue [1] and so are lifestyle-related health problems [2]. Second, partly in response to these changes, the Dutch government [3] obliges organisations in the field of preventive child health care to cooperate more closely with social services, with schools and preschools, forming ‘centres for youth and family’ in order to offer a broad spectrum of facilities to meet the different needs of parents and children. This means that not only doctors and nurses, but also pedagogical and social workers are playing a role in answering questions of young parents about the wellbeing of their children. Questions no longer only concern physical health and growth, but also behaviour, parenting and education. Third, medical doctors have acquired new knowledge and skills during their training to become a specialist in child health care and public health [4], which they scarcely use in current daily practice. Fourth, there is an oncoming shortage of doctors, especially in preventive health care [5], a branch in which only few medical students are interested.

It is important to deal with the combined problem of changing demands from parents and society, changing competences of professionals, and the expected decline in the number of doctors in preventive health care. One possible solution is repositioning nurses in the frontline of the recently formed ‘centres for youth and family’, together with pedagogues and social workers, so they can answer parents’ more general questions, advise on lifestyle issues and help solving parenting and psychosocial problems. A smaller number of doctors will be needed to address more complex medical questions. This fits better with their CANMEDS competences [4]. This solution implies task delegation, i.e. ‘the transfer of authority and responsibility for specific tasks from a person of higher to a person of lower ranking’ [6], and requires specific training for the nurses, so they will be able to perform basic screenings and medical check-ups when needed. There is little experience with task delegation in preventive child health care, but we found a few examples of task delegation to advanced nurse practitioners [7,8]. A Cochrane review [9] showed that ‘appropriately trained nurses can produce as high quality care as primary care doctors and achieve as good health outcomes for patients.’ Abbott states in his book ‘The system of profession’ [10] that highly educated professionals like doctors will only be prepared to delegate tasks if they gain something from it: for example new possibilities and challenges or a lower workload.

In this article we describe an experiment in which trained nurses performed all the regular contacts with selected children from the age of 2 months to 3 years in a well-baby clinic. Overall goal of the experiment was to test feasibility of this working method. We were interested to see whether the nurses were able to do the medical examination, if they actually performed the examination, if they performed it well and how both professionals and parents experienced this working method.

## Methods

The first study question was whether nurses acquired the necessary competences to perform medical examinations in children aged 2 months to 3 years. We expected an improvement in the relevant skills and no change in general nursing competences. The second study question was what percentage of children was actually assigned to the trained nurses (see *‘Intervention’* below). Based on results of a study in Groningen [8, 11], we expected an assignment percentage of 75%. The third study question was whether there was a change in abnormal findings and referrals due to the new working method. From a safety viewpoint, nurses should detect a similar number of abnormal findings as doctors. The fourth study question was how professionals and parents experienced this new working method.

### Study design

A controlled before-after study was carried out. The experiment region consisted of two child health teams, together providing preventive child health care to approximately 3500 children aged 0 to 4 years. Two neighbouring teams, similar in team composition, degree of urbanization and population size and composition, were chosen as control region. All children seen by these teams during the measurement periods were included. Control and experiment regions were compared before and during the experiment as to the development of nurses’ competences, number and type of contacts and abnormal findings and referrals.

### Data collection

In order to answer the question whether nurses acquired the necessary competences, three rounds of questionnaires on competences took place in December 2010, October 2011 and March 2012. The questionnaires (appendix 1) contained items based on general nursing competences and roles [12] as well as items concerning the newly acquired skills in physical examination. Answers were given on a scale with the following response options: 1 (not trained to do this), 2 (trained to do this but never using this skill), 3 (trained to do this and sometimes using this skill) to and 4 (competent to do this and daily using this skill). The questionnaire was especially developed for this experiment and has not been validated yet. Content validity was good because we used an existing and nation-wide used model of nursing competences and roles. Because of logistic problems, the first round of questionnaires was done when training had just started. Therefore nurses of the experiment region were asked to fill in their level of competence before the training. We also collected data on contacts, percentage of children assigned to the trained nurse, and percentage of abnormal findings and referrals from the electronic patient record system. This system, containing information on planning as well as screening results and patient history, had just been fully implemented when the study started. Implementation took place from September 2009 until March 2010. When the study started all teams had implemented this system, but not all teams were consistent in using it. Data on assignment were collected over the period April 2011-March 2012, and contained information on all children aged 0–4 years. Data on abnormal findings and referrals were collected over the period April-Sept 2010 (before the experiment) and over the period April-Sept 2011 (during the experiment). The second data collection period was started when all nurses had been fully trained and were experienced in performing their new tasks. Since registration in the newly introduced electronic patient record system proved not to be uniform in 2010, especially where abnormal findings and referrals were concerned, it was necessary to manually check consistency and correct registration errors. To reduce the workload of these manual data checks, for abnormal findings and referrals only data of newborn babies until the age of 9 months were included in this report. In April 2012 we held focus group interviews with professionals and parents in order to find out about their experiences. Eight nurses, four doctors and five administrative assistants were interviewed in separate focus groups, one for the doctors, two for the nurses and one for the administrative assistants. The remaining two nurses, one doctor and one assistant were asked to read the transcript of the interviews with their colleagues and give additional feedback if they felt it added new information. In the experiment region, three focus group interviews with parents took place, with in total 10 parents attending (two groups of four persons, one group of two persons). In addition to the group interviews 3 telephone interviews were held, and two parents responded by mail when invited for the interview and gave their opinion. Parents were selected based on several criteria: parents with children between 9 and 14 months of age were invited, as we wanted to interview parents who had visited the well-baby clinic several times during the last year. We randomly selected parents and achieved a mix of new parents and parents with older children, who were therefore familiar with the previous working method. The groups were also mixed with regard to the educational level of parents. Both parents and workers were interviewed during a semi-structured interview by an independent interviewer with knowledge of preventive child health care. The interview was based upon a topic list containing different subjects of interest. The topic list had been reviewed beforehand by the research project group consisting of nurses, doctors, managers and researchers. All interviews were conducted in Dutch and the duration of each interview was approximately 90 minutes.

### Statistical analysis

In order to measure the effect of task delegation, the control and experiment regions were compared before and during the experiment. Background characteristics were compared with Chi2 tests. The difference in the change in competences was tested by Mann-Whitney tests, the nurse being the unit of analysis. The difference in the change in abnormal findings and referrals, and the difference in assignment between experiment teams were tested with logistic generalized estimating equations. Assignment was controlled for the age of the child and the clustering of children within nurses (i.e. one nurse assigns many children) [13]. The generalized estimating equations analyses were done with the standard settings of SPSS, using an ‘exchangable’ correlation structure.

Results of the focus group interviews were analysed according to the method of qualitative research described by Boeije [14]: In addition to the interviewer one person (SJB) was present at all interviews to take notes and to record the interviews. All interviews were transcribed verbatim, followed by a selection of keywords linked to the topic list. The keywords were grouped in themes and every interview was analysed on the basis of these keywords, leading to separate reports on experiences of professionals and on experiences of parents. All this was done by the first author of this article (SJB). Both interviewer and interviewee checked the reports for misinterpretations and their remarks were used to make final adjustments.

### Intervention

**Fig 1:**
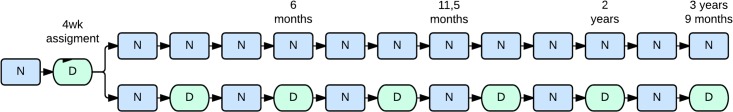
Flow chart experiment schedule (upper row) versus regular schedule (lower row). N = Nurse; D = Doctor

During the experiment children were assigned to one of the following schedules: the experimental schedule where children were seen by a nurse at every contact after the assignment, and the regular schedule (care as usual) where children were seen by doctor and nurse alternately (Fig 1). Assignment was based upon findings of both doctor and nurse: during the home visit when a child was 2 weeks old, the nurse wrote a preliminary advice and when the child was 4 weeks old the doctor physically examined him/her and finally decided on assignment, taking into account the advice of the nurse. The criteria used to assign children to the regular schedule were: pre- or dismaturity (pregnancy <34 weeks or weight <-2SD), birth asphyxia with Apgar scores <7 at 5 minutes; other problems during pregnancy, childbirth or first days of life; chronic disease or congenital malformations; complex psychosocial problems; abnormal findings at 4 weeks from physical examination or developmental tests. If doctor and nurse differed in opinion they discussed this and came to a joint decision. When a child had been assigned to one schedule it was possible to switch to the other schedule. The doctor made this decision, based on professional evaluation of medical risks. If parents did not agree with the experimental schedule, their child was assigned to the regular schedule receiving a special code in the patient record (for analysis purposes only).

The Netherlands School for Public and Occupational Health, a certified educational organisation that also trains doctors to become preventive health care specialists, developed a specific training for this occasion. Prior to the experiment thirteen nurses were trained (five more in a later phase, when additional backup was needed) in performing the required medical examination: screening for dysplastic hip development, amblyopia and congenital heart defects, monitoring of growth and psychomotor development. After 5 training days at school, the nurses continued ‘training on the job’, in their own team, supervised by the doctor of the team. After instruction, the doctors coached the nurses for a period of four months, gradually handing over the new tasks to their colleague nurse. The doctors received a one-day training on how to be a coach and trainer. They also received the instruction to develop new roles. This process started under supervision of a manager and a medical advisor but after the first inventory round, doctors were supposed to continue independently and ask for support when needed.

### Ethics statement

The medical-ethical review board of the UMC Utrecht gave a waiver for formal ethical approval, and also gave a positive opinion for conducting this study (protocol number 10–451), including the informed consent procedure. This procedure was as follows. In regular preventive health care in the Netherlands it is possible to use recorded standard data for anonymized scientific analysis, without additional informed consent. If parents want to make use of preventive health care services, they are informed about working procedures. If this information has been given, it is registered in the digital patient record. For this experiment, all parents in the experiment teams received additional written information. They were asked whether they allowed their child to participate in the experimental schedule. When assignment was about to be made, this was discussed with them. Parents provided their verbal informed consent to participate in this study. If they did not provide informed consent, their child was assigned to the regular care schedule, meaning their child would attend a medical doctor and a nurse alternately. Status of consent to the experiment was registered in the digital patient record. This procedure follows the regular care procedure for asking informed consent for any additional treatment from parents in preventive child health care. Written consent was not obtained as no randomization took place and no additional effort was asked from parents. If parents did not provide informed consent for the experiment, it was still possible to use data from the digital patient record, based on the procedures of regular preventive health care.

## Results

To answer the question on assignment percentage, all assignment data were used from the period April 2011 until March 2012. We included 1997 children. Since we considered a refusal of parents to consent to the experiment condition as a special reason to assign to the regular schedule there were no children excluded based on no consent. Of all parents 26 did not want their child to take part in the experiment. When being asked why they refused, some parents said that they did not feel secure about the adequacy of screening by a nurse. For reasons explained earlier data on abnormal findings and referrals were limited to newborn babies until te age of 9 months. In the experiment region 541 children were included before and 603 during the experiment. The numbers of children included in the control region were 720 and 766 respectively. In Table 1 background characteristics are given. Before the experiment (2010) there was a significant difference in parents’, both mothers and fathers, educational level between experiment and control region, In 2011 this difference disappears, but a significant difference appears in number of children originating from Western countries. In 2011 registration of background characteristics was more complete than in 2010.

**Table 1:** Background characteristics of all children aged 0–9 months in experiment and control region, before (2010) and during (2011) the experiment.

Background characteristics							
		2010			*2011*		
		Experiment	Control	p-value	Experiment	Control	p-value
Number of children	Number of children	n = 541	n = 720		n = 603	n = 766	
Gender	Boys (%)	289 (53,5)	362 (50,3)	0,269	331 (55,2%)	398 (52,0)	0,238
	Girls (%)	252 (46,5)	358 (49,7)		269 (44,8%)	368 (48,0)	
	missing	0	0		3	0	
Country of origin mother	Netherlands	372 (93,9)	299 (92,1)	0,251	553 (92,0)	695 (91,2)	0,010*
	Western countries	8(2,0)	4 (1,2)		8 (1,3)	29 (3,8)	
	Non-western countries	17 (4,3)	22 (6,8)		40 (6,7)	38 (5,0)	
	missing	145	395		2	4	
Country of origin father	Netherlands	375 (95,4)	298 (94,1)	0,493	560 (94,0)	700 (92,7)	0,031*
	Western countries	3 (0,8)	5 (1,5)		6 (1,0)	23 (3,0)	
	Non-western countries	15(3,6)	15 (4,7)		30 (5,0)	32 (4,2)	
	missing	148	402		7	11	
Educational level mother	Low	42 (10,8)	19 (6,5)	0,034*	50 (11,0)	58 (10,1)	0,788
	Middle	152 (39,0)	106 (35,1)		180 (39,5)	221 (38,4)	
	High	196 (50,3)	177 (58,3)		226 (49,5)	297 (51,6)	
	missing	196	418		147	190	
Educational level father	Low	51 (13,3)	36 (12,4)	<0,001*	45 (10,1)	75 (13,2)	0,225
	Middle	160 (41,8)	82 (28,2)		174 (39,1)	201 (35,3)	
	High	172 (44,9)	173 (59,4)		226 (50,8)	294 (51,6)	
	Missing	158	429		158	196	

*Significant difference between experiment and control region.

Educational level of nurses was similar, working experience differed between nurses in the control and experiment teams, the last group being younger with less working experience.

**Fig 2:**
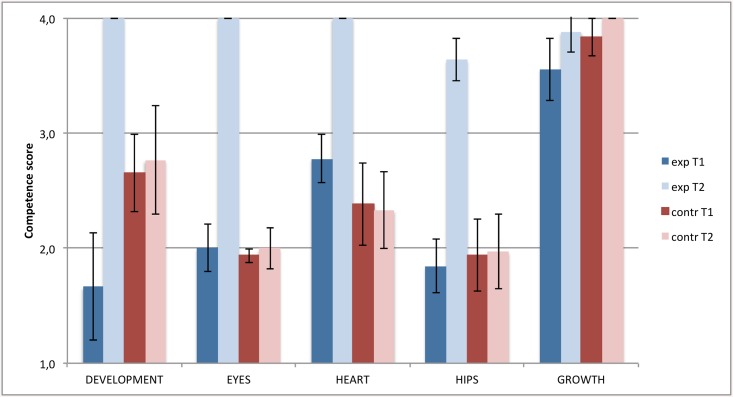
Competence of nurses to perform 5 medical screenings, experiment and control regions compared. T1 = at the start of the training, T2 = after one year experience with the new working method. Exp = experiment region, contr = control region. The 95% confidence intervals are indicated by black error bars.

**Fig 3:**
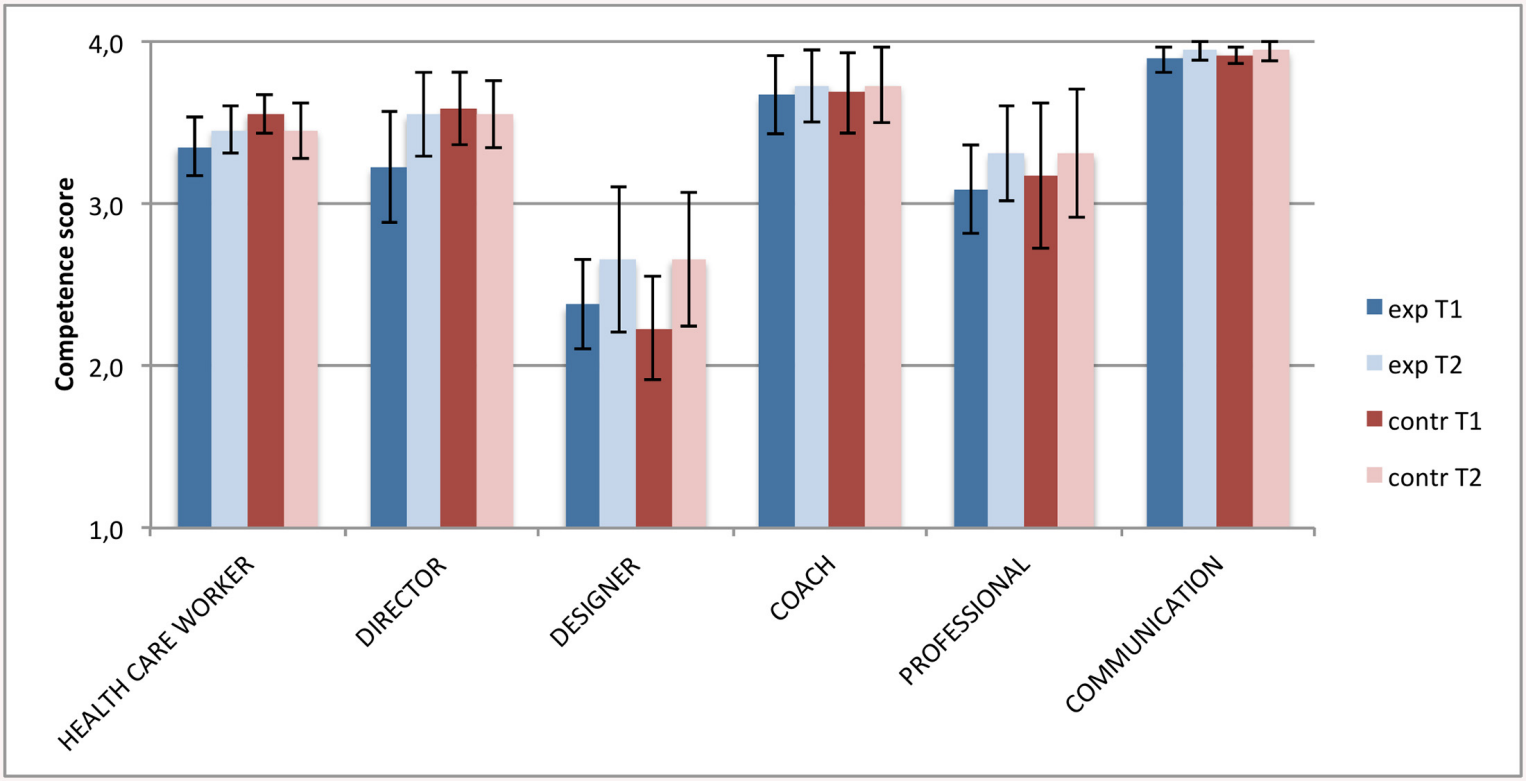
Competence development of general roles nurse, experiment and control regions compared. T1 = at the start of the training, T2 = after one year experience with the new working method. Exp = experiment region, contr = control region. The 95% confidence intervals are indicated by black error bars.

Competency of trained nurses increased for all screening elements except growth, which was already very high (Fig 2). Nurses in the control teams did not change in competence and skills. All differences in change were highly significant (p<0.001) except ‘growth’ (p = 0.7). There were no significant differences in the change in general nursing competence roles (p-values varying from 0.25 to 0.85; Fig 3). At baseline there was a difference between experiment and control nurses in perceived competence for screening on development.

**Fig 4:**
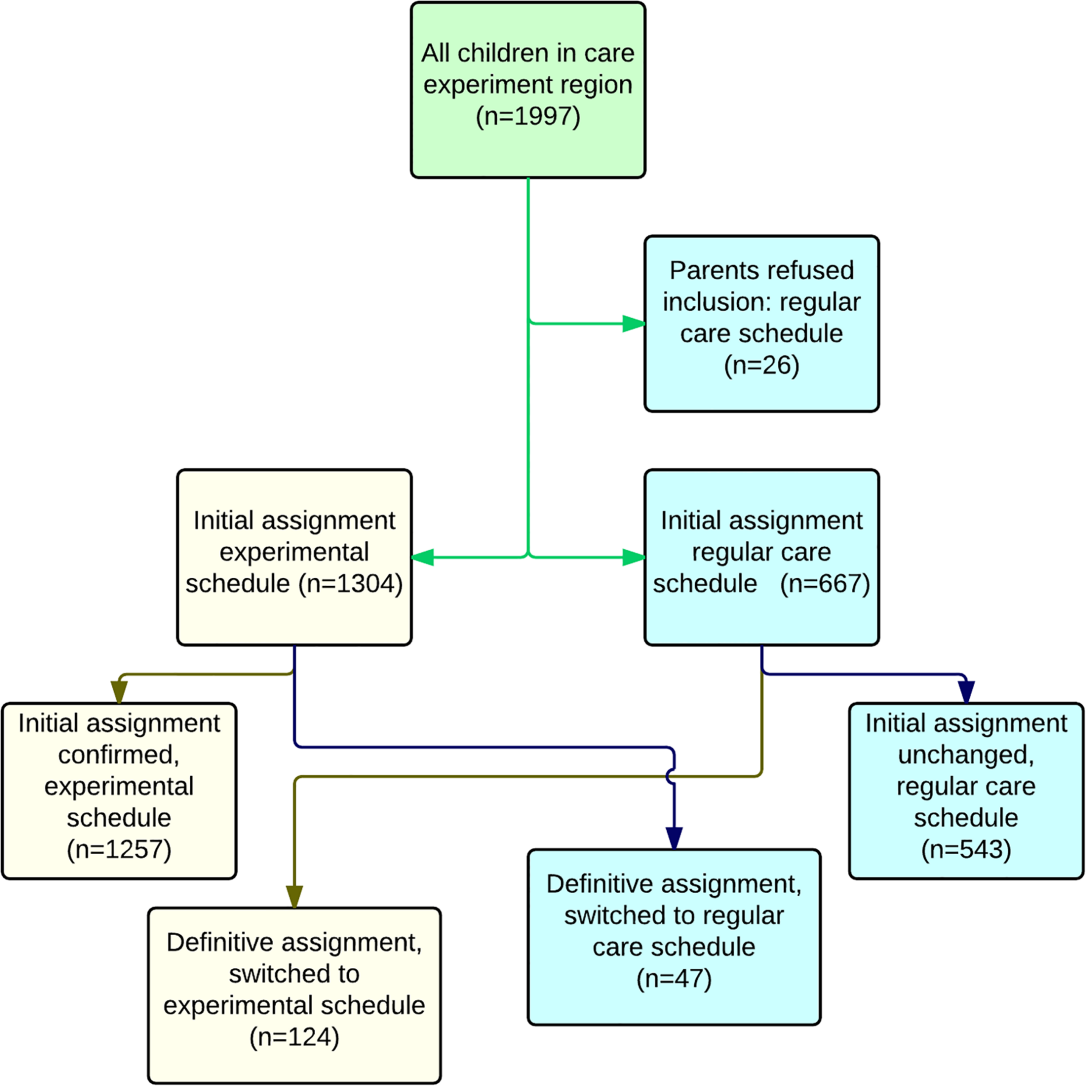
Flow chart assignment procedure, showing number of children assigned to experimental (left) vs regular (right) care schedule.

**Fig 5:**
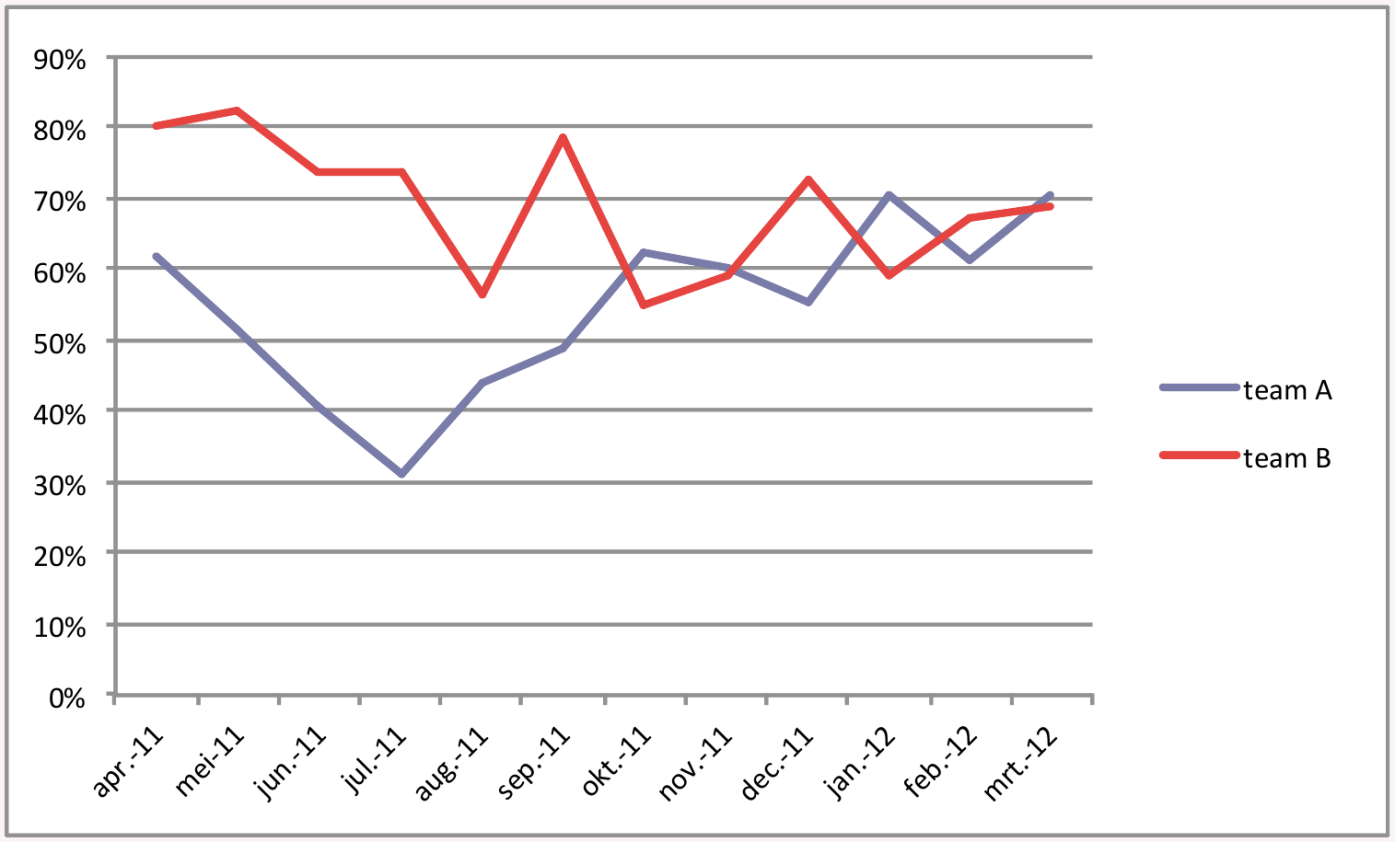
Percentage of children assigned to experiment schedule in both experiment teams.

The second study question was how many children were assigned to the experiment schedule. On average, 69% of the children were assigned to the experiment schedule (Fig 4). The teams changed the assignment of 171 children shortly after a preliminary assignment. We counted only the definitive assignment in calculating assignment percentage. There was a difference between the two experiment teams: team B assigned 77% to the experiment schedule and team A, which was half the size of team B, assigned 60% to this schedule. Assignment percentages were monitored during the experiment, and when there appeared to be a widening gap in percentages between teams, teams were requested to discuss reasons for assignment to the regular care schedule with each other and with the other team. From then on (august 2011) we saw both trendlines gradually change, (Fig 5), converging just below 70%. Overall, the difference in assignment between the teams, controlled for the clustering of children within nurses, was significantly different: odds ratio 2.11 (95%CI 1.34 to 3.32, p = 0.001). The percentage of consultations by the doctor in the control region remained stable during the experiment, but the percentage of consultations by the doctor in the experiment region decreased from 51,6 to 28,8%. This proved to be a significant reduction (p < 0,0001).

**Fig 6:**
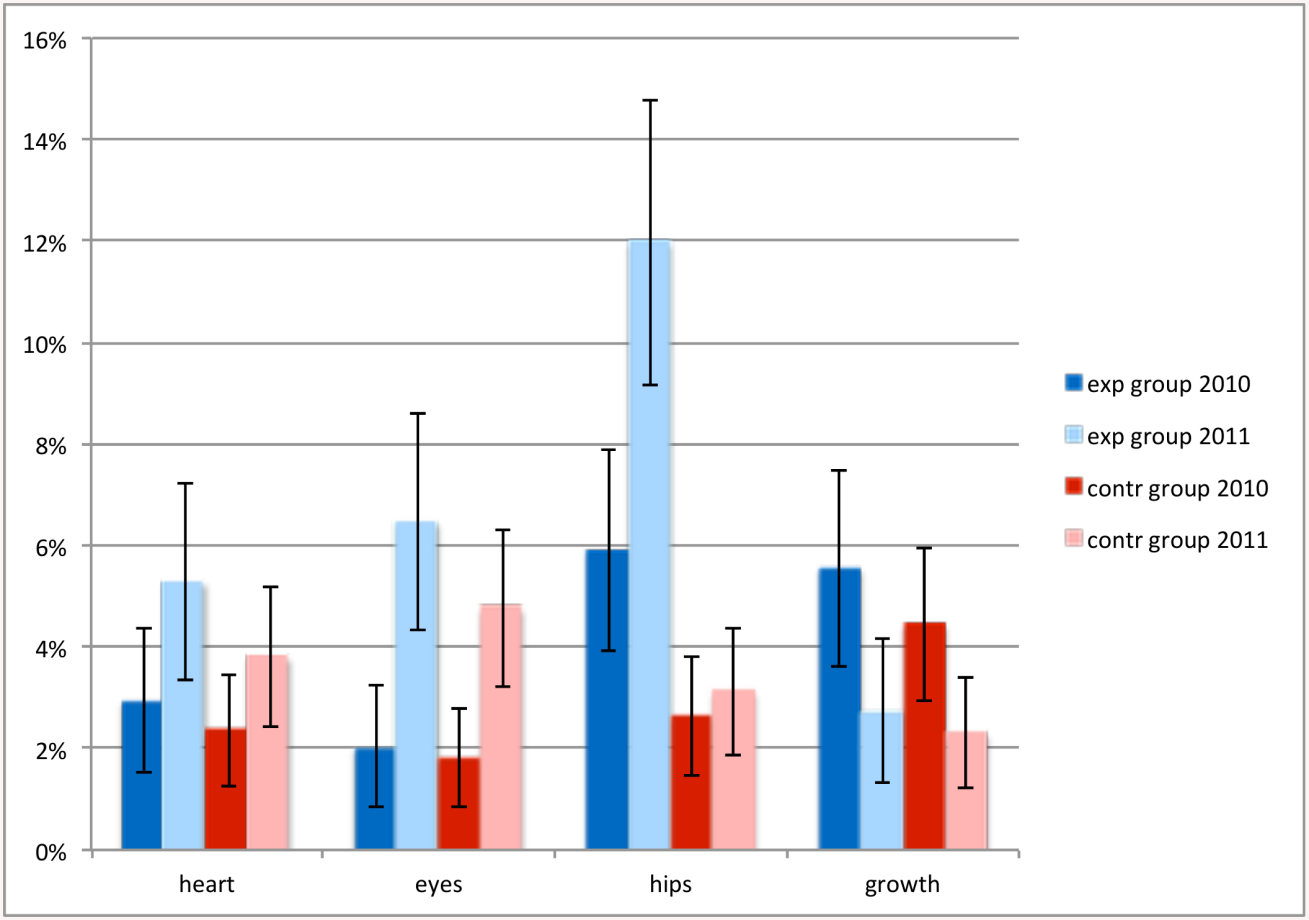
Percentage of children with abnormal findings. A comparison is shown between abnormal findings of heart, eyes, hips and growth, in experiment and control region, before and during the experiment. The 95% confidence intervals are indicated by black error bars.

**Fig 7:**
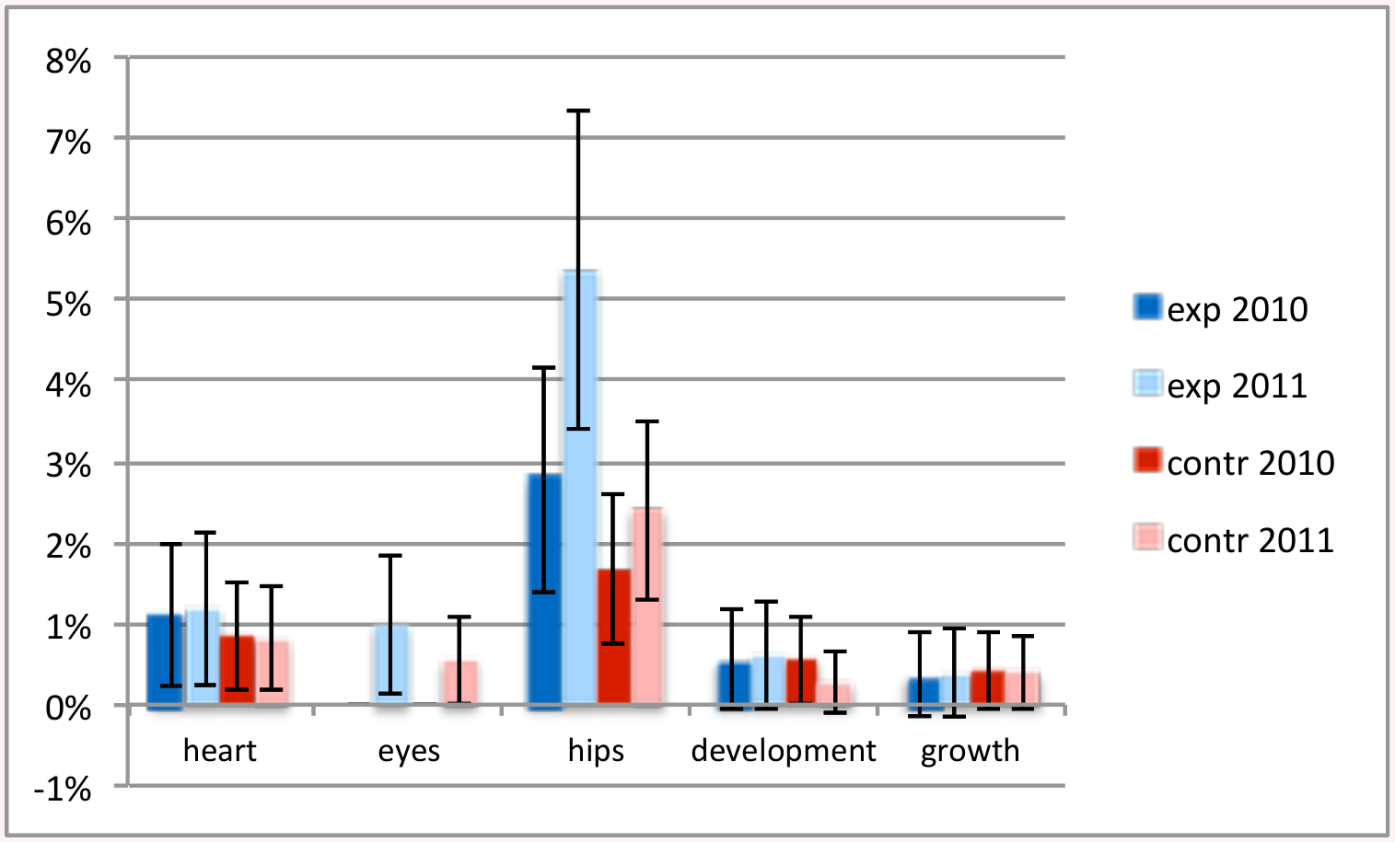
Percentage of children referred with abnormal findings. A comparison is shown between abnormal findings of heart, eyes, hips and growth, in experiment and control region, before and during the experiment. The black error bars indicate the 95% confidence intervals.

The third question was whether there would be a difference in change in the number of abnormal findings and referrals. There was a significant rise in abnormal findings in vision (p = 0.017) and decrease in abnormal findings in growth (p = <0.001) in both experiment and control teams. There was a rise in abnormal findings (p = 0.034) in and referrals (p = 0.035) for hips in the experiment teams, but not in the control teams (Figs 6 and 7). Other referral patterns did not differ significantly between teams or years.

The fourth question concerned the experiences of doctors, nurses and parents. Both doctors and nurses experienced the new method as positive: they felt their job had become more challenging. Nurses experienced they were now getting a more complete picture of children. Doctors felt they had to reflect more on their own actions. They were also confronted with doubts and uncertainties during the process: questions arose about differences between doctors (especially in assessing hips), about the number of doctor consultations needed for every child and about the need for a doctor to be present at all times to be consulted in case of abnormal findings. It proved to be a struggle for the doctors to develop new roles. At the end of the experiment they were able to use 17% of their working hours to perform ‘new tasks’, but most of these tasks were still under construction at the end of the project.

Parents who participated in the focus group discussions felt they were well-informed about the experiment, and they experienced the same level of proficiency and competency with the trained nurse as with the doctor. They met various professionals, especially during the period when the nurses were being trained. Most parents would prefer to have contact with just one or two professionals.

## Discussion

This experiment shows that trained nurses are competent to do medical examinations in infants. Training of child health nurses to perform medical screening in infants results in specific improvement of skills needed to perform these tasks. General nursing competences did not change, as expected. Skills and competences in the nurses of the control teams remained the same. Almost three quarters of all children between 0 and 9 months were assigned to the experiment schedule and examined by a trained nurse.

We observed changes in the number of abnormal findings and referrals, but these changes were not significantly different between experiment and control teams, supporting the assumption that trained nurses performed the examination well. The difference between teams in abnormal findings in hips is discussed below. Interviews showed that both doctors and nurses agreed that the nurses performed the screening procedures correctly, and detected abnormalities well. Both doctors and nurses in the experiment teams thought positively of the new working method, and made some recommendations for improvements. Parents felt well-informed and experienced the same proficiency as before, but less continuity in person, which they regretted. The parents’ positive attitude is in line with findings from a recent review [15], which stated that ‘nurse-led care seems to have a positive effect on patient satisfaction’.

The change in competences of the trained nurses shows that they are capable of performing the newly acquired skills, and is consistent with findings in earlier experiments on task delegation [9, 15, 16]. In their review on doctor-nurse task delegation in primary health care, Laurant et al. [9] stated that ‘findings suggest that appropriately trained nurses can produce as high quality care as primary care doctors and achieve as good health outcomes for patients.’ In the first round of questionnaires however, nurses in the control teams gained a higher score on the item development than their colleagues in the experiment teams whereas they were expected to have similar scores. The explanation for this phenomenon is that the first round took place just after the first training day instead of before. On that day the item ‘development’ had been introduced. All nurses are familiar with part of the examination of development, but due to the instruction the nurses in the experiment teams became aware of a certain lack in their knowledge and skills. They thus became consciously incompetent, one stage further in the ‘four stages of learning’ [17] than their unconsciously incompetent colleagues. In the next round of questionnaires they had acquired the skills to do the development examination in an adequate way and gained higher scores as expected.

Most children could be assigned to the experiment schedule, as expected [8,11] and only a few parents refused to have their child assigned to that schedule. The percentage of assignment differed between teams and also changed during the experiment. The difference between both experiment teams could not be explained by difference in population. Discussing the reason for this difference, both during the process and in focus group interviews, it seemed that a lack of acceptance of the new working method, lack of trust in the competences of the trained nurse and anxiety to hand over responsibilities were the most influential factors on the difference in assignment pattern. Both teams also felt that the criteria for assignment were not completely clear. During the experiment, group sessions were planned with both teams to discuss the assignment process and interpretation of assignment criteria, and gradually assignment percentages became more similar. This illustrates the importance of careful monitoring and evaluation during the process of implementation, in order to achieve the necessary change. [18]

Although we observed no significant difference in the number of abnormal findings or referrals between experiment and control teams (except for hips), it may be possible that the quality of screening differed due to false positive or negative results. It would have been best (since no reference standard is available) if every child had been examined by a nurse and if the outcome had been checked by a doctor who was not a direct team colleague. This could not be realised in this study, so we chose to compare control and experiment team before and during the study. On the subject of congenital hip dysplasia both abnormal findings and referrals were higher in the experiment region. The prevalence of congenital hip dysplasia is around 3–4% in infants 0–6 months, but figures in literature vary greatly [19]. One possible explanation is that the increase in the experiment region lies within the normal expected variation range. Earlier analysis in our organisation showed huge differences between six regions when it came to referral of children on suspicion of hip abnormalities. These differences could not be explained by variation in population characteristics. There was a significant difference in number of children originating from Western countries, but the number of these children is so small that is not influencing the number of hip abnormalities, even if a different prevalence among children from Western countries would be expected. A second explanation is that the screening for hip abnormalities is not distinguishing clearly between normal and abnormal cases. The validity of screening for congenital hip dysplasia by physical examination has proven to be limited, with sensitivity of 86,1%, specificity of 82,3% and post-test probability of 16%. [20]. This means that one out of seven children with a congenital hip malformation will not be detected by screening. A final explanation might be that recent training resulting in very precise physical examination has been leading to more abnormal findings and referrals. Further investigation is needed, following referrals on outcome and to detect false negative findings.

Professionals thought positively of the new working method, but they needed support during the process of implementation and acceptation. Nurses had to learn new skills that gave them more responsibility, which they needed to learn to handle. They needed backup from the doctor who trained them and from their manager to develop confidence in their new role. Doctors not only had to hand over part of their responsibilities, they also had to create their own new role. In Abbott’s [10] words: their ‘system’ was disturbed and they were challenged to find a new role and position. This proved to be a struggle for them, which was unexpected as they are university graduates who have been trained specifically in the field of public health for youth. There seemed to be a lack of competence to initiate renewal and change. We noticed that during the experiment doctors tended to cling to their well-known ‘doctor-like’ tasks, organising extra medical office hours, discussing the need of extra contacts with the doctor for every child and the necessity of a doctor being present all office hours. A possible explanation is that their everyday job has always consisted mainly of standardized and strictly protocolled tasks. Now they were required to become creative thinkers, networkers, and independent professionals, which is more in line with the CANMEDS competences [4], but also poses a serious challenge. When further implementing this working method, both the acceptation process and the competence of developing new roles requires thorough preparation and continuous attention.

Testing feasibility is an important step in the development-evaluation-implementation process [21]. The results of this feasibility study need replication using a more robust study design such as a cluster randomised clinical trial, focussing on effectiveness and efficacy of the working method.

### Limitations

Data from the digital patient files were not completely reliable: the electronic system was relatively new, and this experiment was the first one using huge data extracts from this system. At the start of the first measurement, professionals had just started to register in the digital patient files, resulting in incomplete and non-uniform registration. This becomes clearly visible in the registration of background characteristics: in 2010 pilot and experiment region show other significant differences than in 2011. Incomplete registration seems to be the reason for that change, we therefore consider the data from 2011 as being (more) correct. Referrals could only be counted by reading all individual conclusion text fields in the database. This process may be incomplete because some professionals used other text fields in the patient file to register a referral and did not repeat this in the conclusion text field. Most of these limitations were overcome by manually checking all data from the digital patient files. As described earlier, this limited the number of patients analysed.

## Overall Conclusions

The experiment shows that task delegation from doctor to nurse in preventive youth health care is feasible. It is important to pay attention to the acceptation process of professionals during implementation. Doctors need to learn to hand over their responsibilities and find new challenges, nurses need to take up their new responsibilities. More research is needed to assess effectiveness and efficacy of task delegation in preventive youth health care.

## Appendix 1: Preventive child health care nurses competences questionnaire

### General questions

1. In which team do you work?
  - Elburg/tHarde
  - Putten
  - Zwolle-West
  - Zwolle-Zuid
2. For how many years have you been working as a nurse in preventive child health care?
  - 0–3 years
  - 3–6 years
  - 6–10 years
  - 10–20 years
  - > 20 years
3. For how many hours per week do you work in preventive child health care?
  - Less than 16 hours per week
  - For 16–24 hours per week
  - For 24–32 hours per week
  - More than 32 hours per week

For each of the skills stated below, choose one of the following four categories:

- I have not learned this
- I have learned this, but I do not use it in my work
- I have learned this, and I use it partly in my work
- I have learned this, and I use it fully in my work

NB: It is possible that you have learned skills by experience, not by formal education. This also counts as having learned something

### Caregiver

1. Systematically collecting and registering data
2. Systematically analysing health threats on individual level
3. Systematically analysing health threats on group level
4. Making a care plan with adequate interventions
5. Stimulating an optimal balance between risk factors and protective factors
6. Commencing preventive interventions that influence the environment of children and youth
7. Offering structured information, advice and support concerning health issues, using health information programmes
8. Administering vaccinations
9. Performing screenings to detect abnormalities
10. specific medical screenings:

### Developmental assessment

- Van Wiechen developmental test at 3 months
- Van Wiechen developmental test at 6 months
- Van Wiechen developmental test at 9 months
- Van Wiechen developmental test at 15 months
- Van Wiechen developmental test at 2 years
- Van Wiechen developmental test at 3,9 years

### Physical examination/screening

- Early detection of visual disorders
  - Corneal light reflex
  - Cover test / observation ocular pursuit movements / eye inspection
  - Visual acuity test with APK (Amsterdam Picture Chart)
  - Visual acuity test with Landolt-C
- Screening for congenital heart disorders
  - Inspection of the child during home visit at age 2 weeks
  - Auscultation of the heart
  - General inspection in relation to heart disorders
- Screening for congenital hip dysplasia
- Screening non-descendent testes
- Screening genital malformations
- Examination of body posture and walking pattern
- Detection of skin disorders
- Growth assessment:
  - Length
  - Weight
  - Head circumference
- General examination of:
  - Head and neck
  - Trunk and abdomen

### Director and Designer

1. Initiating and coordinating care for children and youth
2. Referring to other professionals if necessary
3. Initiating and coordinating of and/or participating in preventive programmes
4. Co-developing of new preventive and parenting programmes.
5. Participating in the implementation of nationwide programmes in own region.
6. Contributing to a best-practice nursing policy in preventive youth health care
7. Participating in designing quality care, within institution, region and country

### Coach and Professional

1. Supporting other nurses, assistants and paraprofessionals in performing their own tasks
2. Acting as a role model for trainees and colleague nurses, assisting them with advice and help.
3. Participating actively in innovation of the profession
4. Stimulating professional awareness
5. Contributing actively to expertise development of the profession

### Communication and cooperation

1. Building an effective caregiver-client relationship with parents and children.
2. Maintaining effective relationships with professional partners
3. Connecting to the level and way of thinking of parents and children.
4. Listening actively and collecting information effectively.
5. Discussing health problems in the working area with colleagues in team or in Youth and Family Centre
6. Discussing concerns about an individual child with colleagues in team or in Youth and Family Centre
7. Consultation with other health care professionals
8. Referring to or consulting with the preventive child health care doctor
9. Answering requests for consultation of colleagues
10. Dealing with conflicts of interest and arguments within team or organisation
11. Cooperating with team members
12. Cooperating within the Youth and Family Centre
